# The mediating role of emotions in offline and online political participation: A post-social outbreak study in Ecuador and Chile

**DOI:** 10.3389/fpsyg.2022.1111184

**Published:** 2023-06-09

**Authors:** Loreto Villagrán, Carlos Reyes-Valenzuela, Carolina Alzugaray, Marcos Zumárraga-Espinosa, Jaime Méndez

**Affiliations:** ^1^Departamento de Psicología, Facultad de Ciencias Sociales, Universidad de Concepción, Concepción, Chile; ^2^Universidad Andina Simón Bolívar, Quito, Ecuador; ^3^Escuela de Psicología, Facultad de Ciencias Sociales, Universidad Santo Tomás, Concepción, Chile; ^4^Universidad Politécnica Salesiana, Cuenca, Ecuador

**Keywords:** Chile, Ecuador, emotions, offline and online, political participation, social outbreak

## Abstract

**Introduction:**

In 2019, there was a period of social outbreaks in several Latin American countries, which share a background of social inequality, distrust in authorities, a crisis of representativeness, and discontent towards social and economic policies. In October 2019, in Ecuador and Chile, participation in these protests was characterized by street protests and broad political participation in social networks and alternative media, which were followed or interrupted by the COVID-19 pandemic. These facts have been deeply researched, addressing causal and structural factors of the phenomenon, the alternatives of political participation, and the role of emotions as determinants of action in these contexts. The objective of this study is to explore offline and online political participation (Facebook) after the social outbreak of 2019 in both countries, based on political interest, and how emotions intervene, especially negative ones, in a context of high demobilization.

**Methods:**

A descriptive, correlational ex post facto and cross-sectional methodology was used, with the participation of 367 people, 210 from Ecuador (57.2%) and 157 from Chile (42.8%), aged between 17 and 48 years (M = 22.13, SD = 3.73). The measurement was carried out from 2020 to 2021.

**Results:**

A mediation analysis showed that people who are more interested in politics are more likely to experience anger and anxiety with the political and economic situation, which motivates conventional political participation (Model 1). In Model 2 people who showed greater concern about the political and economic impacts of the COVID-19 pandemic and together with anger, favor online political participation, especially local support.

**Discussion:**

These results suggest the influence of emotions on political participation, which occurs when there is an increase in social discontent due to government policies adopted during the pandemic and which represents a continuity of the discontent that was expressed in the October 2019 social outbreak.

## Introduction

Classic theories of social movements such as relative deprivation (Davis, 1962; Guimond and Dubé-Simard, 1983), social identity model (Reicher, 1996), or the framing model (Gamson, 1992), have at their core the discomfort and political discontent experienced by a population that perceives situations of injustice and social inequality (Tausch et al., 2011). Empirical evidence has shown that discontent is necessary, but not sufficient to explain participation in social movements (Cárdenas and Blanco, 2006). Current evidence suggests that emotions play a determining role in action within these contexts (Jasper, 2011; Poma and Gravante, 2017; Van Ness and Summers-Effler, 2018; Curnow and Vea, 2020; Caruso, 2022). These actions are expressed in different forms of political participation, in which emotions can increase participation, fostering anger and fear, which are related to political discontent and increase the probability of involvement (Valentino et al., 2011).

Research has characterized offline political participation as a real and conventional modality of involvement, and online political participation as an increasingly relevant component to promote political action (Zumárraga Espinosa, 2020). In this context, communication factors and social networks have been crucial at the beginning of social movements (Aravena Lavín, 2022), allowing greater democratization of political participation (Carroll and Hackett, 2006; Coleman and Blumler, 2009; Zumárraga-Espinosa et al., 2017a). On the one hand, it has been found that negative emotions in social networks increase offline participation, in particular, emotions such as anger make participation in protests possible (Min and Yun, 2019). On the other hand, evidence suggests the relevance of online participation through the impact of different online social movements (Hong and Kim, 2021), enabling a form of political expression for groups that are not commonly considered, such as people from low income. It has even been argued that social networks play a key role in the creation of bonds of trust between people participating in social movements (Belli et al., 2020). Likewise, it has been found that people with a greater interest in political issues are more likely to participate in collective actions than those with less interest (Borge et al., 2012), although no significant differences are found in online participation between the same groups (Xenos and Moy, 2007).

### Emotions and their influence on social movements

In general, emotions present a history of agreements and disagreements within the social sciences (Scheff, 1994), despite being present in almost all human behaviors and motivating actions (Izard, 2007; Izard et al., 2008). In recent decades, emotions have been included as an explanatory factor related to different social phenomena (Nardini et al., 2020), even though the idea that emotions represent experiences and not rational processes persists (Jasper, 2011). Concerning social movements, emotions have been approached as transitory reactions to external events or new information (Jasper, 2016). Evidence regarding the role of emotions in the development of social movements mainly shows anger as a central negative emotion in their causal processes (Jasper, 2011, 2014; Nardini et al., 2020). Anger can be expressed as rage and indignation, and interfere with effective strategies to achieve the objectives of collective processes (Jasper, 2016). Complementarily, other emotional reactions are mobilized to reduce political-social discontent, either around anxiety (Best and Krueger, 2011; Wojcieszak et al., 2016), or shame, which could lead to anger (Jasper, 2016). In the field of digital actions Belli et al. (2021), exemplify with young activists in environmental movements how emotions such as fear, facing the planet risk of a future they describe as “apocalyptic,” act as cognitive-emotional frameworks that have fostered activism against climate change.

Social movements have also been associated with positive emotions such as hope (Jasper, 2011; Vilas et al., 2016). Positive emotions would be part of people’s expectation of joining a group, producing feelings of joy and positive affection when participating in group rituals. In particular, hope is interpreted as a means to avoid or modify situations that cause anger or wrath, to reach a better state in the future (Wlodarczyk et al., 2017), or as support to prolong the activity of a group with a specific objective (Sabucedo and Vilas, 2014).

Emotions could also occur in a shared way (“shared emotions”) or reciprocally within the group, where its members would merge into a whole (Jasper, 2014). It has been described that when there is an emotional contagion of emotions, negative reactions can be activated that can have negative effects on individuals (Páez, 2011). Mizen (2015) when analyzing the Occupy movement of 2011 proposed that the emotionality of young people protesting could be analyzed as a single force that influenced group actions and decisions. In the case of emotions in the virtual context, digital emotional contagion has also been described, which would occur in people with shared tastes who can influence each other negatively or positively through publications (Serri, 2018). Studies in the context of the protests in Egypt and Spain (15-M) in 2011, show that the emotion of enthusiasm can be contagious online, generating peaks of enthusiasm and participation, but there is also a risk that after the initial enthusiasm, participation declines (Gerbaudo, 2016). Goldenberg and Gross (2020), agree that due to the amplitude of social networks, the emotional contagion can be very massive but that, for the same reason, there could be an effect of tiredness or fatigue. However, the description of the mechanism of how occurs the phenomenon of contagion (Asún et al., 2021) and when the contagion occurred would be pending (Goldenberg and Gross, 2020). Complementarily, it is also worth highlighting the way in which social networks are used by social movements to emotionally influence the population and achieve support to sustain their actions (Zumárraga-Espinosa et al., 2020).

On the other hand, in the case of the combination of positive and negative emotions, these have been studied through groups that mobilize people to avoid a state of discomfort and approach a pleasant state. Although it is difficult to distinguish between the emotions that are generated when seeking and then when reaching a goal, their importance lies in the fact that they are experiences that accompany the development of a social movement (Jasper, 2014). Hou and Bonanno (2018) conclude that emotions change during a social movement, even if it is short. These authors, in a sample of people who lived through the Umbrella Movement in Hong Kong, found that there was a predominance of negative reactivity accompanied by less positive reactivity during the positive moments of the movement (Hou and Bonanno, 2018).

### Political participation and emotions in the context of a post-social outbreak in Chile and Ecuador

In October 2019, there were days of social protests in both Ecuador and Chile, in the latter, the social outbreak lasted longer. Similar to the global trend, in both contexts, affections and emotions were shared in the face of adverse or deprived situations of people and the struggle to recover citizen public spaces (Belli et al., 2018). Complementarily, both countries were experiencing a strong social polarization that subsequently conditioned preferences in electoral campaigns, in which social networks were profusely used and in which emotions in these contexts were highly potentiated.

Regarding the study of emotions and their influence on social outbreaks, although emotions have been part of the discourses and strategies of current movements in Latin America (see Poma and Gravante, 2017), research in Ecuador and Chile is still incipient and scarce. In the case of Chile, data before the outbreak of 2019 suggest that anger was expressed due to the high cost of living by the middle (53%) and low (45%) socioeconomic classes, and by the actions of the army in both the middle and low classes (44%; González et al., 2021, COES). Similarly, Conejeros et al. (2020), found the existence of a generational gap in fear during the social outbreak, since in younger people predominates the lack of fear and anger in the face of the injustices of the system, while in older people the fear of insecurity and instability predominates, which decrease when seeing the action of the youngest. Asún et al. (2020) found that before the social outbreak, experiencing intense negative emotions of mistrust, worry, and anger was associated with greater social participation. In addition, social participation in the form of attending the protest was related to pleasant emotions such as enthusiasm and hope that lead to a successful perception of the social manifestation. In the case of Ecuador, the limited evidence before the outbreak identified that during the second round of the 2017 presidential elections, positive and negative emotions encouraged political participation in social networks (Zumárraga-Espinosa et al., 2017b) and, during the outbreak, emotions predicted the use of Facebook as a political tool (Zumárraga-Espinosa et al., 2020). However, contradictory evidence has also been found without significant results for emotions as mediators of the relationship between the use of social networks and offline political participation (Zumárraga-Espinosa, 2021).

The context of the COVID-19 pandemic allowed, in both countries, the arrest and interruption of collective processes with a political nature, based on the measures to restrict mobility and association, added to a period of quarantine that lasted until June of 2020 and that counted, in both countries, with strong police and military control. Hence, the actions of mobilization and political participation were scarce at the conventional level, having greater expression in social networks. In both countries, the social outbreak that took place in 2019 was significantly reduced and the context of the COVID-19 pandemic made it possible for online political participation to become the only alternative to protest and express emotions, particularly in the face of the health, political and economic consequences of the pandemic, which although they raised a criticism of the institutions and state representatives, were not necessarily in line with the objectives set out in the October protests. This would also explain why in the few studies of political participation movements and actions in the 2020–2021 period in Ecuador and Chile, emotions have not been reported.

### The present study

This study explores online and offline political participation after the social outbreak of 2019 in Chile and Ecuador, based on political interest and the role of emotions, especially negative ones, in the context of high social demobilization. The context of the study after the October social outbreak overlaps with the COVID-19 pandemic, in which collective political demonstrations were interrupted and emotions of discontent continued to be expressed regarding the state management of the pandemic. This allows us to conclude that social mobilization actions were not deployed in this period, but political participation actions were possible, in some cases, in a conventional sphere (offline) and, more frequently, online. Regarding the latter, the Facebook platform has been specifically considered, because it has been reported as the most used in both countries (Calderón et al., 2021; Santibáñez-Rodríguez, 2022; Zumárraga-Espinosa et al., 2022). Given these considerations, the present study explores the role of emotions in offline and online political participation, based on interest in politics. Because during 2020 and, to a lesser extent, in 2021, it is relevant to know what emotions intervene in political participation in an environment with high restrictions on the rights to assembly, association, and free mobility that were imposed in the countries in the study. The approach to emotions is exploratory and will focus primarily on negative emotions, since the post-outbreak context intensified discontent and social turbulence, with special emphasis on the expression of anger, anxiety, and shame that has been reported. Previously. Therefore, we propose the following hypotheses:

*Hypothesis 1*: The negative emotions of anger and anxiety will intervene in the relationship between interest in politics and offline political participation, in a post-social outbreak context.

*Hypothesis 2*: Negative emotions in general and concern about political, economic and social impacts of COVID-19 will intervene between interest in politics and political participation on the Facebook platform.

## Method

### Design

The study used a descriptive, exploratory, *ex post facto*, correlational, and cross-sectional research design (Ato et al., 2013).

### Participants

The sample corresponded to 367 people, 210 Ecuadorians (57.2%) and 157 Chileans (42.8%), aged between 17 and 48 years (*M* = 22.13; *SD* = 3.73). Regarding gender, 239 are women (65.7%) and 125 are men (34.3%), the vast majority are single (89.4%). Within the sample, 217 participants (59.5%) show an interest in politics, of which 47 (28.5%) self-identify as Left-wing, 219 (60.6%) as Centrist, and 39 (10.9%) as Right-Wing.

### Instruments

#### Interest in politics

Interest in political issues was evaluated through a single item; “How interested would you say you are in politics?,” build upon a Likert-type scale with 4 response options ranging from 1 (not at all interested) to 4 (totally interested).

#### Emotions

The emotions experienced by each participant, based on the current situation in their country, were evaluated. For this, a list of 15 emotions was presented, which vary between joy, hope, pride, enthusiasm, anger, anxiety, resentment, and fear, among others. The reliability of the measure was high for negative (α = 0.90) and positive (α = 0.84) emotions.

#### Concern about the impact of COVID-19

Participants were assessed on their level of concern about the evolution of the COVID-19 pandemic in both countries, and the repercussions it may have on their safety and well-being (Zumárraga-Espinosa et al., 2022). For this, a rating scale was used with response options ranging from 1 (Not at all) to 5 (Very much). Specifically, the concern for the following issues was evaluated: (a) the general economic situation in the country and its possibilities of reactivation; (b) the individual and family economic situation; (c) the response capacity of the hospital system to cases of contagion, and (d) citizen security in their neighborhood and city. The reliability of the measure was high (α = 0.89).

#### Political participation in social media, PPOnline

The degree of political activism developed through Facebook, Twitter, and WhatsApp was measured based on five items, which evaluate activities of expression and political mobilization that can be carried out on digital platforms (Zumárraga Espinosa, 2020). For each social media, a 5-point rating scale ranging from 1 (Never) to 5 (Always) was used, and participants were asked about the frequency of the following political behaviors: (a) writing opinions on issues related to politics on their Facebook’s wall or personal profile [*M* = 1.58, *SD* = 0.84]; (b) comment or respond to political opinions on other people’s walls or pages [*M* = 1.59, *SD* = 0.84]; (c) share images, videos, links and content related to political issues, [*M* = 1.83, *SD* = 1.01]; (d) chat with friends or acquaintances about political issues, [*M* = 1.98, *SD* = 1.05]; (e) mobilize or try to convince other users/contacts to support or join political causes [*M* = 1.21, SD = 0.60], among others. The reliability of the instrument was high (α = 0.89).

#### Conventional political participation, PPOffline

Conventional political participation was evaluated through 14 items which assessed 14 behaviors of conventional political participation, which vary between personally discussing politics with others, contacting a politician or public official to present their points of view, or collaborating with other people from the neighborhood to solve local problems (Zumárraga Espinosa, 2020). A 5-point rating scale was used, ranging from 1 (I have never done it and would never do it) to 5 (I have done it many times), and participants were asked about the frequency of the following political behaviors: (a) Collaborate with the neighbors or people from near sectors to solve local problems [*M* = 2.25, *SD* = 0.89]; (b) Attend legal and peaceful marches, demonstrations and collective mobilizations [*M* = 2.59, *SD* = 1.30]; (c) Attend a meeting or political debate, [*M* = 2.19, *SD* = 1.03]; (d) Personally discuss politics with others, [*M* = 2.64, *SD* = 1.23]; (e) Participate in strikes or unauthorized demonstrations [*M* = 2.02, *SD* = 1.19], among others. The reliability of the measure was high (α = 0.88).

### Procedure

This research complied with ethical research principles at national and international levels. The participants, mostly college students, were directly contacted and explained the objectives of the study, the anonymous and confidential nature of their responses, and the possibility of withdrawing from the study at any time they wish. The participants were contacted by professors from three universities, one in Quito (Ecuador) and two in Concepción (Chile). Two versions of the questionnaire were created, an online version through the Google Forms platform, which was sent *via* email to each participant for them to respond voluntarily within 2 months. The paper version was answered mainly by the participants from the universities in Concepción. In both countries, the questionnaire contained informed consent, a section where sociodemographic data could be completed, and the items of the scale about the concern of COVID-19, negative emotions, and offline and online political participation. The data collection was carried out in two periods of time: in November 2020 in Chile and in January and February 2021in Ecuador.

### Data analysis

A descriptive analysis was carried out to identify the frequency of negative emotions and offline and online political participation. Descriptive statistics, including mean, standard deviation, skewness (S), kurtosis (K), Cronbach’s alpha, bivariate correlations of all study variables, and mean comparisons (t statistic) were calculated using SPSS-25 software. Subsequently, two mediation analyzes were carried out, using the PROCESS macro for SPSS: in the first one, model 4 (Hayes, 2017) was used, to verify if negative emotions such as anxiety, anger, and shame are mediators between interest in politics and offline political participation. A second mediation model, specifically through model 6, evaluated the relationship between interest in politics and political participation on Facebook, through intervening variables such as negative emotions and concern about the consequences of COVID-19. In both analyses, indirect effects were obtained using a bootstrap procedure, based on 10,000 samples and 95% confidence intervals correcting for bias.

## Results

### Descriptive statistics

Regarding the descriptive analysis (Table 1), statistically significant correlations were found in both countries between the study variables with the emotions of anxiety and anger (Table 2). On the other hand, significant differences are found when comparing the means by country in two measures: anxiety *t*(365) = 7.211, *p* = 0.000, *d* = 0.76, and offline political participation, *t*(361) = 7.211, *p* = 0.000, *d* = 0.51.

**Table 1:** Descriptive statistics of the study variables.

	Ecuador (*n* = 210)					Chile (*n* = 157)				
	*M*	*SD*	*S*	*K*	α	*M*	*SD*	*S*	*K*	α
Interest in politics	2.64	0.89	−0.22	−0.67	-	2.65	0.81	−0.21	−0.41	-
Anger	3.04	1.36	0.12	−1.24	-	3.07	1.29	−0.05	−1.13	-
Anxiety	2.80	1.26	0.26	−0.92	-	3.72	1.13	−0.64	−0.33	-
COVID-19 concern	19.5	5.15	−0.87	−0.18	0.94	19.6	3.56	−0.55	−0.01	0.79
PP offline	25.1	7.66	0.63	0.04	0.87	29.1	9.57	0.71	0.38	0.89
PP online (Facebook)	16.9	5.74	1.12	1.73	0.84	17.2	7.76	1.64	2.97	0.89

M, Arithmetic Mean; SD, Standard Deviation; S, Skewness; K, Kurtosis; α, Cronbach’s alpha.

**Table 2:** Bivariate correlations of the study variables by country.

	Ecuador (*n* = 210)		Chile (*n* = 157)	
	Anxiety	Anger	Anxiety	Anger
Interest in politics	0.22^**^	0.11^ṭ^	0.04	0.18^**^
COVID-19 concern	0.28^**^	0.34^**^	0.37^**^	0.36^**^
PP offline	0.29^**^	0.20^**^	0.24^**^	0.35^**^
PP online (Facebook)	0.27^**^	0.19^**^	0.18^ṭ^	0.22^*^

ṭ marginal.

* *p* < 0.05;.

** *p* < 0.01.

### Mediation model

**Figure 1:**
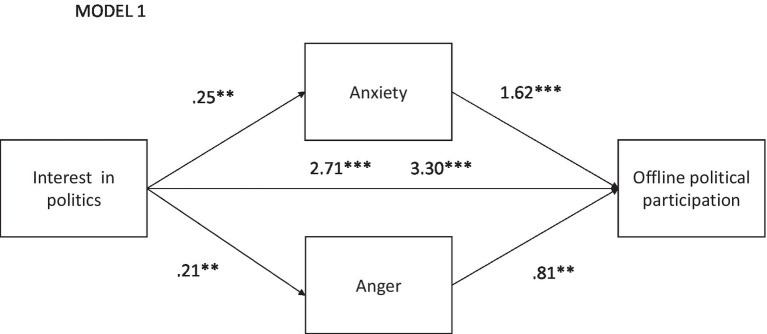
Relationship between interest in politics and PP offline mediated by anxiety and anger. **p < 0.01 and ***p < 0.001.

A mediation analysis was carried out, using the PROCESS macro for SPSS and model 4 (Hayes, 2017). First, the role of emotions as mediating variables between IP and offline political participation (PPOff) was contrasted. The results presented in Figure 1 confirm the significant relationship between IP and PPOff (B = 2.71, t = 5.46, *p* = 0.000). As mediating variables, two emotions presented significant effects; anxiety with IP (B = 0.25, t = 3.22, *p* = 0.002) and PPOff (B = 1.64, t = 4.59, *p* = 0.000), and anger with IP (B = 0.21, t = 2.65, *p* = 0.008) and with PPOff (B = 0.81, t = 2.36, *p* = 0.018). Based on the effects of the mediating variables, the significance between IP and PPOff is maintained (B = 3.30, t = 6.43, *p* = 0.000), with an R^2^ = 10%. This is evidence of partial fulfillment of hypothesis 1, concerning the mediating effect of the emotions of anger and anxiety, but which rules out the emotion of shame.

**Figure 2:**
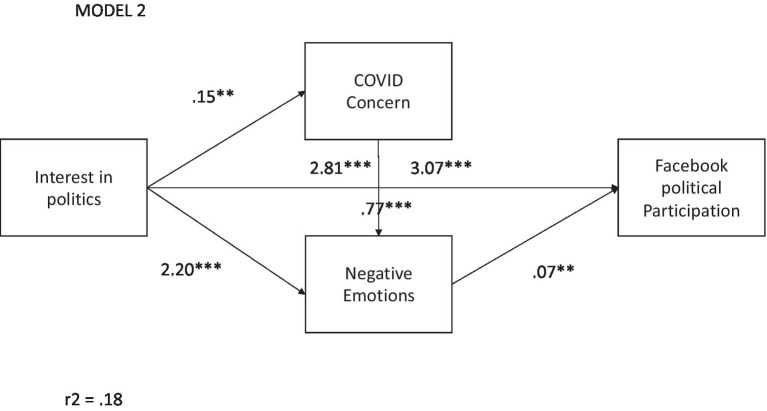
Relationship between interest in politics and PP online (Facebook) mediated by concern about the impacts of COVID-19 and anger. **p < 0.01 and ***p < 0.001.

For the second hypothesis, a mediation analysis was carried out using model 6, to evaluate the mediating role of emotions and concern about COVID-19 (CC) in the relationship between IP and online participation in Facebook (PPF). The results presented in Figure 2 identify a significant relationship between IP and PPF (B = 2.91, t = 7.53, *p* = 0.000). As mediating variables, significant effects of CC were found with IP (B = 0.83, t = 2.69, *p* = 0.007) and not significant with PPF. A second variable, negative emotions, had a significant relationship with IP (B = 0.18, t = 2.22, *p* = 0.026), with CC (B = 0.09, t = 5.71, *p* = 0.000), and with PPF (B = 0.64, t = 2.41, *p* = 0.016). From the effects of the mediating variables, the significance between IP and PPF is maintained (B = 3.08, t = 8.06, *p* = 0.000), which suggests a double path that uses CC and negative emotions and a second one only with negative emotions and PPF, with an R^2^ = 18%. Thus, hypothesis 2 is confirmed, regarding the role of the negative emotions and concern for COVID-19 as an intervening factor in political participation on Facebook.

### Discussion

The aim of this paper is to study offline and online political participation in Ecuador and Chile in a less explored period of their recent political reality, located after the social outbreaks of October 2019, and coinciding with the COVID-19 pandemic. Due to the sanitary restrictions imposed by the governments on people at this stage, it is of interest to explore what individual and collective actions are taking place in this context. At this point, it is relevant to know how negative emotions intervene in people with a certain interest in politics and enable participation, in a demobilizing context. Although the measures implemented allowed greater state control of the protests, it is also clear that in the period after the social outbreaks, emotions continued to be present in state and civil society reactions, discourses, and/or strategies.

Our findings suggest that offline participation is driven by emotions such as anger and anxiety, which is consistent with previous research that reveals the activation that these types of emotions promote. At this point, it is worth asking whether these emotions are necessarily “negative” or “mobilizing” (Poma and Gravante, 2017) since their expression would be more related to ways of individual coping in a political, economic, and health context that presents different needs than those of the social protest of 2019. On the other hand, it is argued that the study of emotions and their intervention in political actions do not assume a fixed and stable position, in the face of emotional expressions that in politics are more uncertain and that show states of connection and disconnection against political information. In addition, the consideration of emotions in an abstract way reduces the possibilities of addressing the context in which they arise and how they are processed by individuals in contexts of high emotional diffusion. At this point, the notion of emotional contagion is key to understanding how, in the face of information with a high negative emotional charge (i.e., such as that experienced in the post-flood and health emergency context), faster and more powerful emotional, behavioral and cognitive responses are generated, and how digital platforms have facilitated this exchange.

Regarding hypothesis 1, results suggest that anxiety and anger are emotions that intervene in offline political participation, this would explain why people interested in politics are more likely to experience these two emotions, which are related to the socio-political situation of each country. In this sense, political participation behaviors are mainly related to talking about politics with others, generating spaces for discussion, and organizing face-to-face actions to help others or to protest against the country’s situation. Concerning anxiety, since it is related to fight or flight decisions, it could be assumed that this emotion gives rise to vigilance and reactive action. Likewise, it can be identified that anxiety was reinforced in a pandemic context, which limited bonds, mourning processes and reinforced the consideration that human relationships are being configured in spaces of uncertainty and anxiety (Belli, 2022). Although the empirical support for the role of anxiety in the formation of political participation is contradictory, the findings presented here support the idea that anxiety increases conventional participation (Marcus et al., 2000; Becker, 2014). In this case, anxiety manifests as a means to reduce the discomfort that the situation in the country is generating, specifically, through the neighborhood and local actions, given the impossibility of generating a more widespread action. These actions even contradict the restrictive measures established by the authorities and this support allows people to maintain closer and face-to-face contact, especially with neighbors. The latter is in line with the assertion that, in crisis situations, there is a need to share emotional experiences with others and to influence the emotional states of others (Rimé, 2009).

In the case of anger, it has been suggested that it presents a direct relationship with feelings of political discontent and social unrest (Valentino et al., 2011), which allows greater sensitivity to social problems and a more active commitment to seek face-to-face political participation channels (Van Zomeren et al., 2012). It has also been suggested that anger can be a mobilizing emotion since it would encourage struggle or resistance, although this needs to be demonstrated in each context (Poma and Gravante, 2017). Here, it has been considered that the interaction between anxiety and anger would allow greater personal involvement in political affairs and it even manifests in calls for collective actions that were limited in the period analyzed.

Concerning the fulfillment of hypothesis 2, it is evident that online political participation on Facebook activates by concern about the consequences of the pandemic and by negative emotions. This suggests that people who are more interested in politics are more likely to worry about the political, economic, and social impacts of COVID-19 since they have a broader vision of the country’s situation and are more aware of the consequences, dangers, and risks that such a health emergency entails. This concern is not situated solely on an emotional level but represents a cognitive assessment since this can trigger emotions, as reported in a study carried out by Zumárraga-Espinosa et al. (2022). This assessment leads us to reflect that there is a threatening situation that must be resolved (Rico et al., 2020; Dettano and Cena, 2021) and that together with a poor state management, triggers negative emotions, which activates political participation on Facebook. It is important to mention that, in digital media, participation is low cost, and such consistent and long-term motivations are not required to channel these threats through this social network (Muñiz and Corduneanu, 2021; Zumárraga-Espinosa et al., 2022). In this way, political participation by Facebook questions and criticizes the actions that the states are not carrying out to reduce this threatening situation (Arancibia Aguilera, 2021; Marquez Domínguez et al., 2021; Ntontis et al., 2022; Pérez Sánchez and Solís, 2022; Zumárraga-Espinosa et al., 2022) and which is expressed in different levels of emotional contagion (Belli, 2022).

Taken together, our findings show that, in contexts of high uncertainty and concern such as the pandemic, people with a political interest generated certain conditions to cope with the emotional contagions expressed in social media and channel the expressions through political participation in one of the spheres explored (Zumárraga-Espinosa et al., 2020). In addition, they carry out a political reading of the situation, which maintains emotional continuity with respect to the polarized political climate and instability (Metzler et al., 2022). social and economic situation that each country had been presenting since the social outbreak of October 2019. These findings come to counter the idea that actions of political nature were completely demobilized, but were mostly redirected to a virtual space; in other cases, they remained on a conventional plane, associated with talking about the needs that people presented in the context of the pandemic. Thus, during the years prior to the pandemic in which various adversities occurred that congregated demonstrations and protests, and were related to negative emotional expressions, such as those explored in this study, regarding the situation in the country (i.e., anxiety, worry, and anger). These emotions allowed sustaining the discussion of structural political issues that were being analyzed and questioned in the October 2019 protests and would be related to the reappearance of social protests in Ecuador and Chile in 2022, although with less intensity.

### Limitations and future lines of research

The study has limitations to consider. First, the collective actions that individuals and groups carried out during this time were not evaluated, which could have been of greater interest than just exploring political participation. This implies collective actions better express collective motivations, emotions, and convictions that were present in the protests of October 2019. However, given that the period evaluated was characterized by several restrictions on the rights of assembly, association, and protest, the possibilities to investigate these actions were more limited than addressing political participation. A second limitation lies in the absence of more cognitive variables, such as politicized identity and collective efficacy that have been confirmed in previous research on social manifestations. Possibly, the inclusion of these variables could shed light on how people maintained or modified the objectives that mobilized them in the 2019 protests. Despite this, a greater role for emotional expressions was chosen for this period, since it was more difficult to mobilize collectively and the conditions only allowed individual actions, which were more exposed to various emotions, given the reactions and discourses that were implanted in the period of the pandemic.

In addition, the sample selected in the study is oriented toward a sector that does not allow a generalization of the results and that, on the contrary, could present greater resources for political participation. Although university students indeed participated in the October protests, it is not possible to generalize the results even among the university population of Ecuador and Chile, given the diversity of actors and actions that this group carried out in October and the subsequent period. The main goal was to present how university students participated politically in a demobilizing context that did not offer the possibility to participate in decisions about the pandemic and academic activities.

The results regarding positive emotions should be interpreted with caution, since it is not possible to ensure that they are not present in the development of the movements studied. It is necessary to investigate whether they could have appeared or accompanied the negative emotions at some other time that this study did not consider, or even appear at a future time. Furthermore, it is worth asking about the emotional management and different emotional expressions of the participants, during the pandemic during 2020, characterized by a highly threatening emotional climate with fear and permanent risks to life. Focusing on emotions such as anxiety and anger, which in theory promote action, could also be explained in another way, since it was important to know whether these emotions generated a paralyzing effect in the above-mentioned context.

Finally, we recommend that future studies include an analysis of the periods of greatest and least activity in social protest and not only address emotional expressions or actions displayed in active periods of social protest. It is important to identify intervening variables in periods of lesser mobilization that could generate subsequent outbreaks, as occurred in Ecuador in June 2022. At this point, the application of explanatory models of social protest such as the SIMCA (Social Identity Model of Collective Action), could incorporate a broader analysis regarding the role played by emotions in the intermediate stages of the emergence of offline political participation and/or collective protests. Finally, future research could investigate the influence of people’s emotions within environments with a negative emotional climate and how that stimulates other forms of resistance, not necessarily expressed in collective actions or social outbreaks.

## Data availability statement

The raw data supporting the conclusions of this article will be made available by the authors, without undue reservation.

## Ethics statement

The studies involving human participants were reviewed and approved by the Comité de Ética y Bioética del Departamento de Psicología de la Universidad de Concepción. The patients/participants provided their written informed consent to participate in this study.

## Author contributions

LV: data analysis and text writing. CR-V: data analysis and text writing and translation. CA: text writing, translation, and formatting. MZ-E: data analysis and text writing. JM: data collection and formatting. All authors contributed to the article and approved the submitted version.

## Conflict of interest

The authors declare that the research was conducted in the absence of any commercial or financial relationships that could be construed as a potential conflict of interest.

## Publisher’s note

All claims expressed in this article are solely those of the authors and do not necessarily represent those of their affiliated organizations, or those of the publisher, the editors and the reviewers. Any product that may be evaluated in this article, or claim that may be made by its manufacturer, is not guaranteed or endorsed by the publisher.
